# Exploration of treatment strategies for cerebral cavernous malformations: two case reports on non-resection treatment and literature review

**DOI:** 10.3389/fonc.2025.1513254

**Published:** 2025-01-28

**Authors:** Yibo Han, Dong Liang, Jing Guo, Yibao Wang, Yong Wang

**Affiliations:** ^1^ Department of Neurosurgery, The First Affiliated Hospital of China Medical University, Shenyang, Liaoning, China; ^2^ Department of Organ and Tissue Anatomy, Hamamatsu University School of Medicine, Hamamatsu, Shizuoka, Japan; ^3^ Department of Optical Neuroanatomy, Hamamatsu University School of Medicine, Hamamatsu, Shizuoka, Japan; ^4^ Imaging technician in Charge, Shenyang Renren Kangye Hospital, Shenyang, Liaoning, China

**Keywords:** cavernous malformations, midbrain, hydrocephalus, endoscopic third ventriculostomy, case report

## Abstract

**Background:**

Cavernous malformations are common vascular abnormalities of the central nervous system, but cavernous malformations of the cerebral aqueduct are rare. The choice of treatment is influenced by various factors.

**Case Description:**

We report two cases of midbrain cavernous malformations. Both cases involved midbrain lesions obstructing the cerebral aqueduct, leading to obstructive hydrocephalus. The primary symptoms and complaints of the patients were related to hydrocephalus. Prior to surgery, patients underwent comprehensive imaging evaluations and received endoscopic third ventriculostomy rather than tumor resection. Both patients had favorable recoveries. We also reviewed the literature and discussed the choice of treatment strategies.

**Conclusion:**

Cavernous malformations are slow-progressing central nervous system lesions with a relatively benign natural course. When selecting a treatment strategy, clinicians should carefully consider the underlying cause of the patient’s primary symptoms and the specific objectives of the surgery. Avoiding overly aggressive resection that fails to address the main symptoms and potentially causes irreversible damage is crucial.

## 1 Introduction

Cerebral cavernous malformations (CCMs) are common vascular malformations of the central nervous system. CCMs occur in both sporadic and familial forms, frequently affecting young adults, typically between the ages of 20 and 50 (1–3). The sporadic form is generally associated with a single isolated lesion, while the familial form is linked to multiple lesions and mutations in three specific genes: CCM1, CCM2, and CCM3. Familial cerebral cavernous malformations (FCCM) are inherited in an autosomal dominant manner due to heterozygous mutations in one of these three genes, with approximately 40-60% of FCCM cases being attributed to this mode of inheritance (4–6). The natural course of cavernous malformations is relatively benign, with about 21% of patients remaining asymptomatic. However, depending on the location and size of the lesions, patients may present with clinical symptoms such as seizures, headaches, neurological deficits, and intracerebral hemorrhage (7–9). Most cavernous malformations are supratentorial, with the incidence of midbrain CCMs being around 9%-35% (10, 11). Here, we report two rare cases of midbrain aqueductal CCMs and discuss them in the context of a literature review.

## 2 Materials and methods

### 2.1 General data

Retrieve and collect the complete treatment and follow-up data of patients with deep cerebral cavernous hemangiomas, who presented primarily with symptoms related to hydrocephalus, treated in our hospital from January 2024 to August 2024.

### 2.2 Literature review

To gather relevant literature, we conducted a search in PubMed for English-language articles published between 2002 and 2024 using the Boolean search terms: “(cavernous angioma OR cavernous malformation OR cavernous hemangioma OR cavernoma OR cerebral cavernous malformations) AND (brain OR cerebral OR intracranial OR brainstem).” A total of 5,135 related papers were retrieved. We specifically selected adult CCM cases located in the midbrain and adjacent structures (midbrain, thalamus, and third ventricle) that provided detailed descriptions of patient conditions, lesion locations, treatment plans, and outcomes. Considering that resective treatment is undoubtedly the first-choice therapy in certain cases,we excluded cases involving acute hemorrhage, mass effect leading to specific neurological deficits, or other conditions severely impacting quality of life. Finally, we selected 14 relevant articles, including a total of 15 patients for the literature review.

## 3 Case report

### 3.1 Case 1

**Figure 1:**
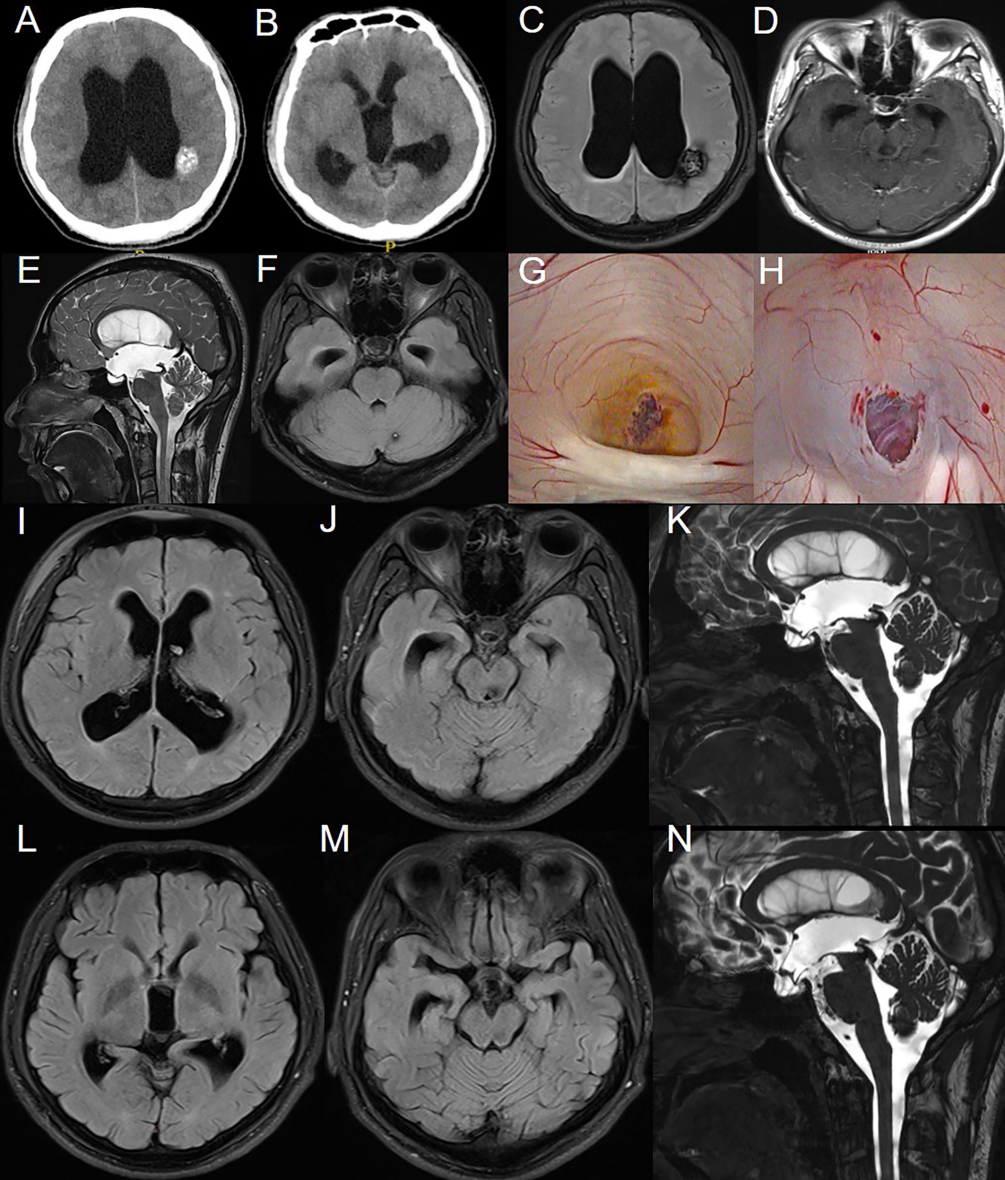
Preoperative imaging examination, intraoperative findings, and postoperative follow-up of the patient in Case 1. **(A-F)**: CT and MRI reveal multiple intracranial masses and clear signs of obstructive hydrocephalus. **(G)**: CCM obstructing the cerebral aqueduct with surrounding hemosiderin deposition. **(H)**: After balloon catheter puncture and fistula creation, the fistula was patent, and the basilar artery showed good pulsation. **(I-K)**: MRI results on the third day postoperatively. **(L-N)**: MRI results at the third-month postoperative follow-up.

The patient is a 21-year-old male with a long history of headaches accompanied by vision decline and a past history of seizures. Over the past two months, he experienced significant worsening of his vision. Fundoscopic examination revealed normal intraocular pressure in both eyes, papilledema, and optic atrophy. No other significant abnormalities were detected on physical examination. MRI scans (**Figures 1A–F**) showed supratentorial ventricular enlargement, periventricular interstitial edema, and a flattening of the sulci and cisterns. Multiple intracranial lesions appeared isointense on T1-weighted imaging and exhibited mixed hyperintense and hypointense signals on T2-weighted imaging, without enhancement. These lesions displayed patchy short T1 signals internally and surrounding ring-like hypointense signals. The entrance of the midbrain aqueduct was obstructed, with downward displacement of the third ventricle floor, empty sella syndrome, and shallowing of the pontine and basal cisterns. Using cine phase-contrast MRI, cerebrospinal fluid (CSF) flow velocities at the membrane structure above and below the midbrain aqueduct were assessed (**Supplementary Video S1**), indicating complete obstruction of the aqueduct.

An endoscopic third ventriculostomy (ETV) was performed using a flexible neuroendoscope to establish a CSF shunt, opening the prepontine cistern. Intraoperative ventricular exploration was consistent with chronic hydrocephalus (**Figures 1G, H**), showing a fenestrated septum pellucidum, enlarged foramen of Monro, downward displacement of the third ventricle, and anterior displacement of the lamina terminalis. The cavernous malformation obstructing the entrance to the midbrain aqueduct was clearly identified, with hemosiderin deposits indicative of prior hemorrhage. A third ventriculostomy was performed under the microscope, with fenestration of the Liliequist’s membrane in the basal cisterns, and a robust pulsation of the basilar artery was observed through the stoma.

Follow-up MRI on postoperative day 3 (**Figures 1I–K**) showed improvement compared to preoperative imaging, with decreased ventricular enlargement and deepening of the cerebral sulci and cisterns. The floor of the third ventricle was elevated, and the empty sella syndrome was alleviated, with the stoma remaining patent. This was further confirmed by cine phase-contrast MRI (**Supplementary Video S2**).

At the 3-month postoperative follow-up MRI (**Figures 1L–N**), the stoma remained patent, with further reduction in ventricular enlargement and resolution of periventricular interstitial edema. Cine phase-contrast MRI demonstrated clear CSF flow from the third ventricle floor to the prepontine cistern (**Supplementary Video S3**). The patient reported significant relief from headaches, with no seizure episodes observed since surgery, although there was no notable improvement in vision.

At 11.5 months post-surgery, a telephone follow-up was conducted. The patient’s headache symptoms had resolved, and there have been no seizures to date. Regarding vision impairment, the patient regularly attends a rehabilitation center for treatment and reports slight improvement in vision compared to before.

### 3.2 Case 2

**Figure 2:**
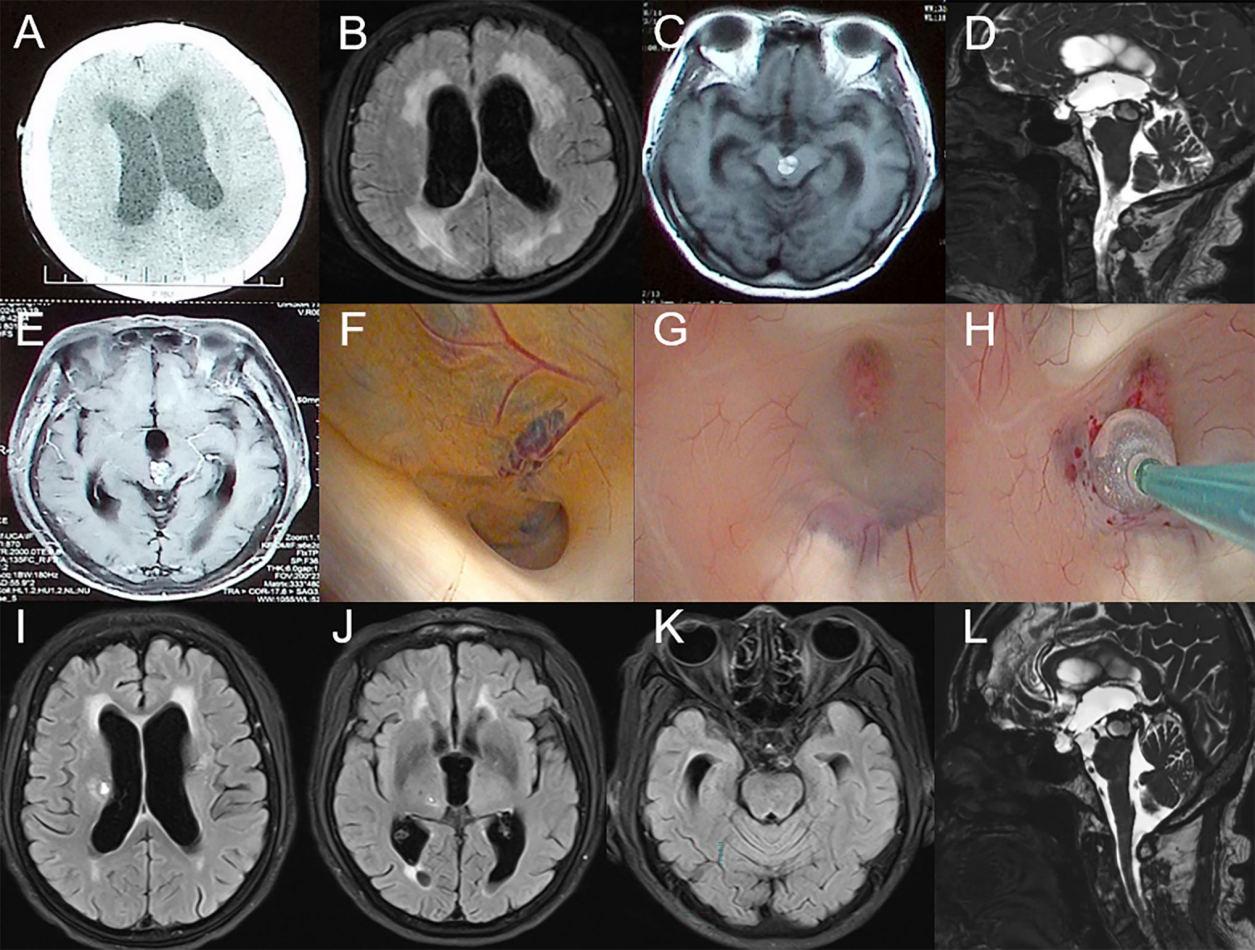
Preoperative imaging examination, intraoperative findings, and postoperative follow-up of the patient in Case 2. **(A-E)**: CT and MRI reveal a mass in the midbrain and clear signs of obstructive hydrocephalus. **(F)**: CCM obstructing the cerebral aqueduct with surrounding hemosiderin deposition. **(G-H)**: Select the appropriate site and create the fistula. **(I-L)**: MRI results on the seventh day postoperatively.

The patient is a 74-year-old female who had recovered from a midbrain hemorrhage treated conservatively nine years ago, with no residual neurological deficits. Eight months ago, she began experiencing multiple episodes of transient loss of consciousness followed by falls, with no recollection of the events afterward. Approximately one and a half months ago, she developed gait instability and gradually worsening cognitive function. About a month ago, she started experiencing urinary incontinence, which progressed to fecal incontinence ten days before admission. On physical examination, the patient was found to have anisocoria with the left pupil measuring 3.5 mm in diameter and non-reactive to light, while the right pupil was 3.0 mm in diameter and reactive to light. She exhibited left ptosis, with the left eye in an abducted position and restricted adduction. Muscle strength was graded 4/5 in both upper limbs and 3/5 in both lower limbs. CT scans (**Figure 2A**) showed bilateral ventricular enlargement with a mixed-density mass in the brainstem obstructing the midbrain aqueduct. MRI (**Figures 2B–E**) revealed shallow supratentorial sulci and cisterns, ventricular enlargement, downward displacement of the third ventricle floor, and a mixed signal mass in the brainstem, with localized narrowing and obstruction of the midbrain aqueduct, consistent with obstructive hydrocephalus.

An endoscopic third ventriculostomy (ETV) was performed, creating a stoma at the floor of the third ventricle to communicate with the prepontine cistern. Intraoperative findings (**Figures 2F–H**) were consistent with hydrocephalus, showing ventricular enlargement and downward displacement of the third ventricle. The midbrain aqueduct was obstructed by a cavernous malformation, with hemosiderin deposits indicative of prior hemorrhage. A third ventriculostomy was created, and the Liliequist’s membrane in the basal cisterns was fenestrated, with robust basilar artery pulsations observed through the stoma.

Follow-up MRI on postoperative day 7 (**Figures 2I–L**) showed improved ventricular enlargement, with deepened sulci and cisterns and reduced interstitial edema compared to preoperative imaging. The floor of the third ventricle was elevated, and the stoma remained patent. Cine phase-contrast MRI (**Supplementary Video S4**) confirmed these findings. The patient’s hydrocephalus-related symptoms were significantly alleviated compared to preoperative status, with reported relief of pyramidal tract-related symptoms. She was subsequently transferred to a rehabilitation facility for further treatment.

At 5 months post-surgery, the patient’s symptoms had significantly improved. Compared to one-week post-surgery, urinary and fecal incontinence had completely resolved, cognitive function was normal, and the patient was able to walk independently. Imaging results were consistent with expectations (**Supplementary Figure S1**).

## 4 Literature review

A total of 14 articles, encompassing 15 cases, met these criteria (12–25), with details summarized in **Table 1**. In these cases, nearly all patients presented with hydrocephalus caused by CCM mass effect, with no specific neurological deficits directly attributable to the tumor itself. Among the 15 reported cases, 7 patients received treatment solely targeting hydrocephalus symptoms, including endoscopic third ventriculostomy (ETV) and ventriculoperitoneal (VP) shunting. Of these, 6 cases achieved favorable outcomes with significant symptom improvement, while 1 case had a poor prognosis due to postoperative hemorrhage within hours after surgery. In Case 5, although surgical resection was performed, only the thalamic lesion among multiple CCMs was removed for diagnostic purposes. The midbrain lesion causing hydrocephalus was left untreated and managed instead with ETV to address the hydrocephalus. Excluding Case 5, 6 cases involved CCM resection. Among them, Cases 9 and 15 underwent simultaneous CCM resection and ETV, both achieving good outcomes. However, 4 patients showed no significant postoperative improvement and even had poor prognoses. Notably, Case 4 required a second surgery for VP shunting due to postoperative hydrocephalus. Additionally, one patient with multiple CCMs presenting with vertigo achieved satisfactory outcomes and long-term follow-up through conservative management alone.

**Table 1:** Summary of reported cases of CCM which located in the midbrain and adjacent structures.

Case ID	Authors/References	Age (gender)	Primary symptoms	Localization	Therapy	Follow-up (months)	Outcome
1	Bulluss, K J et al., 2004 (13)	43, F	headache, nausea	dorsal midbrain	VP shunt	30	Improved
2		47, M	headache	left thalamus	ETV	28	Improved
3	Cristini, Alejandro et al., 2004 (12)	24, M	headache, drowsiness, gait instability	midbrain tectum	EVD, surgical resection	7	Improved
4	Darwish, B et al., 2005 (14)	47, F	sudden onset of tinnitus, self-resolved	third ventricle	surgical resection, VP shunt	NA	Improved
5	Giannetti, Alexandre Varella. 2013 (15)	56, M	confusion, gait and visual acuity disturbances	midbrain, dorsal thalamus	ETV, surgical resection	28	Improved
6	Feletti, Alberto et al., 2016 (17)	62, F	progressive headache, memory loss, gait instability, urinary incontinence	midbrain aqueduct	ETV	12	Improved
7	Belousova, Olga B et al., 2017 (16)	34, F	confusion, disoriented, up-lateral right eye deviation, vertical gaze palsy, ataxia, bilateral edema of optic nerve discs	midbrain, right frontal lobe, left temporal lobe	ETV	60	Improved
8	Kulason, Kay O et al., 2017 (18)	52, F	anterograde amnesia	third ventricle	surgical resection	4	Worsened
9	Li, Jiuhong et al., 2021 (19)	57, F	headache, dizziness	third ventricle	ETV, surgical resection	3	Improved
10	Loh, Daniel De-Liang et al., 2022 (20)	62, F	insidious onset short-term memory loss, unsteady gait, urinary incontinence, left-sided dysaesthesia	midbrain aqueduct	surgical resection	3	Unchanged
11	Hyo−Jeong Lee et al., 2023 (22)	47, F	disequilibrium	fourth ventricle	surgical resection	3	Worsened
12	Raghad 24 (24)	37, F	confusion, diplopia, dysarthria, left-sided weakness	pons	VP shunt	0.8	Worsened
13	Wei Yang et al., 2024 (25)	74, F	unresponsiveness, lethargy, nausea, vomiting	midbrain aqueduct	VP shunt	NA	Improved
14	Nur ‘Afeena Al Fahmi Abdul 21 (21)	40, M	vertigo	left frontal lobe, right temporal lobe, right parietal lobe, cerebellopontine angle	Conservative management	NA	Improved
15	Shuang 23 (23)	29, M	headache, diplopia	dorsal midbrain	ETV, surgical resection	3	Improved

VP shunt, Ventriculoperitoneal shunt; ETV, Endoscopic Third Ventriculostomy; EVD, External Ventricular Drainage. NA: No information Available.

In conclusion, for CCMs located in the midbrain and adjacent structures, neurosurgeons should exercise caution when choosing treatment options. The literature review suggests that the benefits of resective treatment for tumors in such conditions are relatively limited. In contrast, treatments targeting the primary issue, hydrocephalus, through approaches like ETV, often result in better prognoses.

## 5 Discussion

Based on imaging examinations and intraoperative endoscopic exploration, both cases were ultimately diagnosed as cavernous malformations (CCMs). Case 1 involved multiple lesions, while Case 2 featured a solitary lesion in an elderly patient. In both cases, the midbrain lesions obstructed the cerebral aqueduct, leading to obstructive hydrocephalus. The progression of hydrocephalus was gradual, with significant dilation of the third ventricle. In Case 1, the patient’s improvement in vision progressed slowly post-surgery. We believe this is due to the gradual progression of the patient’s hydrocephalus, which differs from the effects caused by acute intracranial pressure elevation in the short term. Therefore, the treatment of obstructive hydrocephalus alone did not show immediate effectiveness. However, the imaging results and the complete resolution of the patient’s headache symptoms also confirm the efficacy of our treatment. In Case 2, despite the obvious mass effect causing corticospinal tract symptoms, such as ptosis and restricted eye movement, the primary cause of the patient’s symptoms was obstructive hydrocephalus, which significantly impacted the patient’s quality of life. Recent studies suggest that conservative treatment also has a positive impact on CCM (26). In a recent cohort study involving 265 CCM patients treated conservatively with a follow-up period of at least 6 months, the results showed that most conservatively treated CCM patients did not experience symptomatic hemorrhage during the follow-up, and few required intervention, with death due to CCM being rare (27). Additionally, for symptomatic or recurrently hemorrhaging brainstem CCMs, surgical treatment is an important option (28). In conclusion, for our patient, choosing endoscopic third ventriculostomy (ETV) to treat obstructive hydrocephalus effectively alleviated the patient’s symptoms while avoiding the potential harm caused by surgery itself. This approach is both reasonable and meaningful.

The diagnosis of familial multiple cavernous malformations (FCCM) requires meeting one or more of the following criteria: (1) presence of multiple CCMs (≥5); (2) at least two family members diagnosed with CCM; (3) mutation in one of the three genes associated with FCCM (29). Typically, CCMs appear as “popcorn-like” lesions with mixed high and low signals on T1- and T2-weighted MRI sequences (30). The MRI appearance of CCMs is influenced by the time interval since hemorrhage, leading Zabramski et al. to classify CCMs into four types (2). Although CCMs are generally considered to have a relatively benign natural course, some patients may experience focal neurological deficits, which can sometimes be irreversible (1, 31, 32).

In recent years, cohort studies on surgical treatment of CCMs have increased, with treatment options including traditional craniotomy, neuroendoscopic surgery, gamma knife, and laser interstitial thermal therapy, all showing favorable outcomes (33–36). For incidentally discovered, asymptomatic CCMs, conservative management—primarily periodic monitoring considering the patient’s age and lesion location—is generally preferred over immediate surgical resection (37–40). The timing of surgical intervention for symptomatic CCMs, especially those causing neurological deficits, remains controversial (11, 41, 42). This is particularly true for midbrain CCMs, where some reports suggest that aggressive surgical treatment does not significantly increase the risk of adverse outcomes (43–45). For intraventricular CCMs, although studies have indicated a high hemorrhage propensity (46), the resection of CCMs in the fourth ventricle or nearby areas carries significant risks (17). This is particularly unacceptable for patients whose primary symptoms are hydrocephalus or those who are asymptomatic. Therefore, when making treatment decisions, factors such as the size, location, mass effect, and surgical risks associated with the CCM must be carefully considered. For symptomatic CCMs located in superficial brain areas, or for patients with recurrent symptomatic hemorrhages even when the lesion is deep-seated, surgical resection is a reasonable choice (47, 48).

Certainly, this study also has objective limitations. Most notably, the sample size is small, with only two patients. This is a retrospective study rather than a standard cohort study or a controlled trial. Factors such as the surgical skills and decision-making ability of the lead surgeon may also influence the generalizability of the final results. Furthermore, the lack of preoperative quality-of-life assessments for the patients further limits the conclusions of this study. Future research will focus on larger-scale prospective studies, with comprehensive quality-of-life indicators and longitudinal follow-up, to better understand the impact of surgery on patient outcomes and improve clinical strategies in future studies. Finally, we also hope for multicenter studies, especially those including diverse nationalities and ethnicities, as this will provide more generalizable and meaningful results for this type of research.

## 6 Conclusion

As a slow-progressing central nervous system lesion with a relatively benign natural course, the treatment strategy for CCMs is influenced by various factors. While the hemorrhage risk of CCMs cannot be overlooked, overly aggressive surgical approaches may cause greater harm to patients. Our cases and the literature review offer new perspectives and insights into surgical decision-making, contributing to better treatment outcomes and prognosis. By demonstrating the effectiveness of addressing hydrocephalus as the primary cause of symptoms rather than pursuing aggressive surgical resection of cavernous malformations, this study supports a more conservative treatment approach in similar cases. This approach not only reduces the risk of potential surgical complications but also emphasizes the importance of individualized patient care. Clinicians are encouraged to carefully evaluate the primary cause of symptoms and consider less invasive options when appropriate. This is particularly important when dealing with younger patients or elderly patients, where optimizing treatment outcomes while preserving quality of life is especially crucial. The patients we reported on benefitted from the surgeries and have been followed up long-term, further supporting the value of this conservative approach.

## Data Availability

The original contributions presented in the study are included in the article/**Supplementary Material**. Further inquiries can be directed to the corresponding author.

## Supplementary material

Supplementary Figure 1 Follow-up imaging results of Case 2 at 5 months post-surgery.

Supplementary Video 1 Preoperative cerebrospinal fluid (CSF) cine phase-contrast MRI of Case 1.

Supplementary Video 2 Postoperative day 3 cerebrospinal fluid (CSF) cine phase-contrast MRI of Case 1.

Supplementary Video 3 Postoperative 3-month cerebrospinal fluid (CSF) cine phase-contrast MRI of Case 1.

Supplementary Video 4 Postoperative day 7 cerebrospinal fluid (CSF) cine phase-contrast MRI of Case 2.
