# Supplementary figures and images for: Exploration of treatment strategies for cerebral cavernous malformations: two case reports on non-resection treatment and literature review
Source: Front Oncol. 2025 Jan 28;15:1513254. doi: 10.3389/fonc.2025.1513254 (PMC11811082; doi:10.3389/fonc.2025.1513254)

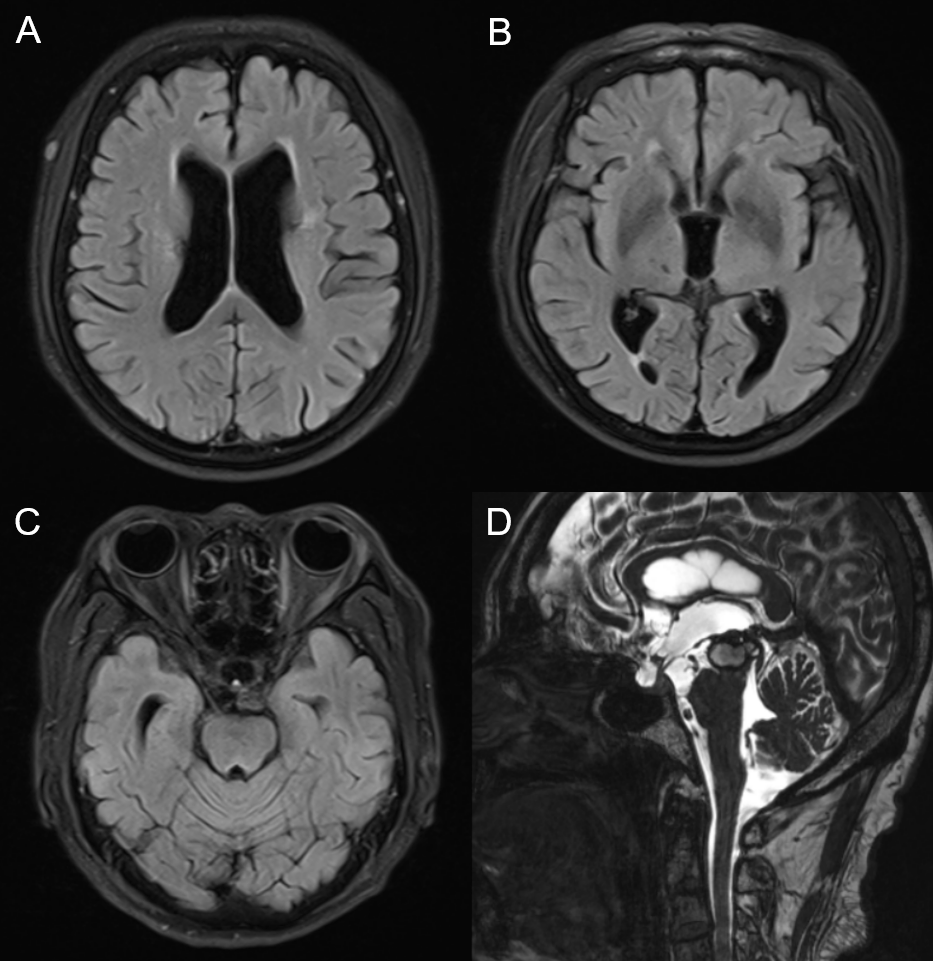

Supplement: Supplementary Figure 1 — Follow-up imaging results of Case 2 at 5 months post-surgery. [file Image1.tif]
